# Talar Dome Investigation and Talocrural Joint Axis Analysis Based on Three-Dimensional (3D) Models: Implications for Prosthetic Design

**DOI:** 10.1155/2019/8634159

**Published:** 2019-11-07

**Authors:** Da-Hang Zhao, Di-Chao Huang, Gong-Hao Zhang, Yun-Ping Fan, Jian Yu, Shao-Bai Wang, Kan Wang, Xin Ma

**Affiliations:** ^1^Department of Orthopedics, Huashan Hospital, Fudan University, Shanghai, China; ^2^Department of Orthopaedics, Ruijin Hospital, Shanghai Jiaotong University School of Medicine, Shanghai, China; ^3^Department of Traumatic Orthopaedics, Ningbo No. 6 Hospital, Zhengjiang, China; ^4^Shanghai InnoMotion Inc., Shanghai, China; ^5^Key Laboratory of Exercise and Health Science of Ministry of Education, Shanghai University of Sport, Shanghai, China; ^6^Department of Radiology, Huashan Hospital, Fudan University, Shanghai, China

## Abstract

Ankle joint kinematics is mainly stabilized by the morphology of the talar dome and the articular surface of tibiofibular mortise as well as the medial and lateral ligament complexes. Because of this the bicondylar geometry of talus dome is believed to be crucial for ankle implant design. However, little data exist describing the precise anatomy of the talar dome and the talocrural joint axis. The aim of this study is to document the anatomy of the talar dome and the axis of the talocrural joint using three-dimensional (3D) computed tomographic (CT) modeling. Seventy-one participants enrolled for CT scanning and 3D talar model reconstruction. All the ankles were held in a neutral position during the CT scanning. Six points on the lateral and medial crest of the talar dome were defined. The coordinate of the six points; radii of lateral-anterior (R-LA), lateral-posterior (R-LP), medial-anterior (R-MA), and medial-posterior (R-MP) sections; and inclination angle of the talar dome were measured, and the inclination and deviation angles of the talocrural joint axis were determined. The mean values of R-LA, R-LP, R-MA, and R-MP were 19.23 ± 2.47 mm, 18.76 ± 2.90 mm, 17.02 ± 3.49 mm, and 22.75 ± 3.04 mm. The mean inclination angle of the talar dome was 9.86 ± 3.30 degrees. Gender variation was found in this parameter. The mean inclination and deviation angles were 8.60 ± 0.07 and 0.76 ± 0.69 degrees for the dorsiflexion axis and −7.34 ± 0.07 and 0.09 ± 0.18 degrees for the plantarflexion axis. Bilateral asymmetries between the medial and lateral crest of the talar dome were found, which resulted in different dorsiflexion and plantarflexion axes of the talocrural joint. Currently, no ankle implants replicate this talar anatomy, and these findings should be considered in future implant designs.

## 1. Introduction

Quantitative investigations of the complex morphology of the talar dome, which articulates with the tibial plafond and mainly conducts coupled motion with the rotational component of dorsiflexion and plantarflexion, are crucial for understanding the joint function and designing ankle implants [1–5]. The main articular surface of the current ankle implant is between talar component and polyethylene (PE) insert. The design features of current ankle implant are quite different, and there exist some failures of total ankle arthroplasty (TAA) resulted from poor prosthesis designs [6–8].

It was controversial on the radius of talar dome morphology and the direction of the talocrural joint axis from the published studies. Although superior surface of the talar dome was typically pulley shape with a central mild concaved surface and two asymmetrical borders [9], early studies suggested that the trochlear surface could be regarded as a frustum, in which the apex directed medially and the rotation axis coincided with the line connecting the medial and lateral malleolus tips. Furthermore, the medial border of the talar dome had a smaller radius of curvature than that of lateral border [10, 11]. And these observations have been copied in the design of some ankle systems [12–14]. However, the concept of the fixed rotation axis of the talocrural joint contradicted with the coupled behavior of ankle joint complex, and several later results in vitro or vivo [4, 15–18]. Some recent research studies revealed that the talar dome was a saddle-shaped, skewed, truncated cone with laterally oriented apex and the radius of curvature of the lateral border was smaller than the medial border without the fixed rotation axis [19–21], which were different from previous conclusions [10]. Furthermore, the unilateral and bilateral asymmetries between the radii of the medial and lateral talar trochleae were found by dividing the trochleae into anterior and posterior regions, in which the anteromedial radius was smaller than anterolateral or posteromedial radius [22]. These results were similar to one early study that medial trochlea had two parts of different radii, whereas the lateral trochlea had one radius [23].

Certain amount of error might arise by current methods for talar measurement. On the one hand, intrinsic error could exist when measuring cadaver specimens or manually determining subject points on the three-dimensional (3D) reconstructed model. Thus, the coordinate system established by the landmarks might not be accurate [10, 11, 22–25]. On the other hand, not many studies positioned the foot and lower extremity in the neutral position, in which both the longitudinal axis of the second metatarsal and mechanical axis were parallel to the sagittal plane of gantry during CT image collection [26].

The purpose of this study was to investigate (1) the relative distribution of circles on the lateral and medial crests of the talar dome, (2) the radii of circles, and (3) the talocrural joint axis based on 3D reconstructed model scaling and alignment.

## 2. Materials and Methods

### 2.1. Study Participants

Eighty-four healthy participants registered initially. Medical history was recorded to exclude the participants with previous trauma. Two of the authors (DZ and GZ) evaluated the bilateral ankle X-ray independently to exclude the participants with deformity or degenerative changes. The protocol was approved by the Ethics Committee of Huashan Hospital, Fudan University. Seventy-one participants enrolled in this study including 56 males and 15 females. The mean age was 25.17 (21 to 37). The mean height, weight, and body mass index (BMI) were 173.72 ± 6.22 cm, 68.48 ± 11.16 kg, and 22.62 ± 2.94. A male participant, whose height (172 cm) and weight (67 kg) were most close to the mean value, was selected as the reference participant. Finally, one reference and 70 target participants were included.

### 2.2. Reconstruction of the 3D Models

Computed tomography (CT) images of all the participants were taken from 10 cm proximal to the tibiotalar joint to the sole in our institution (Brilliance iCT, Philips, Cleveland, Ohio, USA) using axial slices (120 kV, 250 mA, slice thickness = 0.67 mm, slice increment = 0.67 mm, and pixel size = 512 × 512 matrix). Digital Imaging and Communications in Medicine (DICOM) images were imported into Mimics (Version 9.0, Materialise NV, Belgium). After proper segmentation, filtering, and rendering, 3D talar models were obtained with stereo lithography (STL) format. All the models were then imported into Geomagic Studio 2013 (Geomagic, Morrisville, North Carolina, USA) to refine the geometry. As left and right tali show a strong degree of symmetry [27], we randomly selected one side for CT scanning. Finally 34 right and 37 left feet and ankles, of which right side was selected for the reference participant, were included.

### 2.3. Position of the Reference Participant during CT Scanning

**Figure 1:**
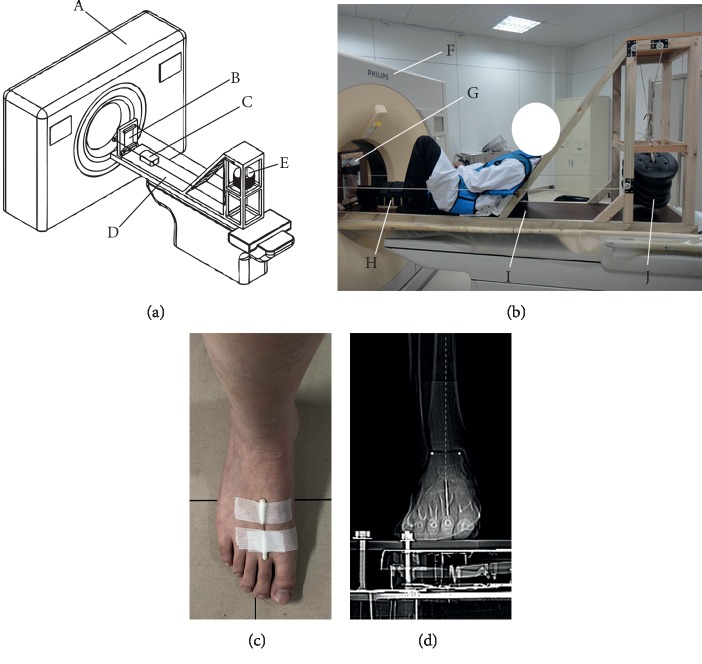
Foot-loading device was used when CT scan was performed on the reference participant. (a) The main device included a CT gantry (A), a foot plate with stress sensor (B), a radiolucent pad (C), a frame (D), and a loading control system (E). (b) The reference participant was sitting on the foot-loading device which included a CT gantry (F), a foot plate with stress sensor (G), a radiolucent pad (H), a frame (I), and a loading control system (J). (c) A 5 cm Kirschner wire (2 mm Φ) was marked along with the axis of the second metatarsal by the landmark on the skin. (d) Making sure the extension line of the Kirschner wire passes through the midwidth of the talar dome and coincides with the mechanical axis during CT scanning.

Right foot and ankle CT images were collected when half weight-bearing was loaded on the reference participant using a foot-loading device, which was used in our published studies [28, 29]. The device includes a foot plate with stress sensor, a frame, and a loading control system (Figure 1(a)), in which the foot plate is parallel to the coronal plane of CT gantry and the frame is parallel to the ground. The right foot sole of the reference participant was placed on the foot plate with left knee in flexion. The right leg was supported by a radiolucent pad to make it perpendicular to the foot plate, and then half weight was loaded on the reference participant by the loading control system (Figure 1(b)). A 5 cm length Kirschner wire (2 mm Φ) was marked along with the axis of the second metatarsal (Figure 1(c)). During CT image collection, the foot and ankle were positioned in the neutral position by making sure the extension line of Kirschner wire passing through the midwidth of the talar dome simultaneously coincides with the mechanical axis (Figure 1(d)).

### 2.4. Model Scaling, Alignment, and Coordinate System Established

**Figure 2:**
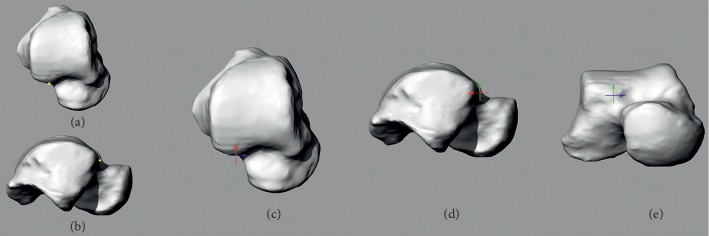
Coordinate system. (a) Origin of the coordinate system was observed from top view. (b) Origin was observed from lateral view. (c) *X* and *Y* axes were perpendicular to sagittal and coronal planes. (d) *Y* and *Z* axes were perpendicular to coronal and transverse planes. (e) *X* and *Z* axes were perpendicular to sagittal and transverse planes.

First, left models were transferred to right using left-right mirror transform. Next, a uniform scaling approach was used to quantify the volume of target models to the reference model, and then target models were aligned to the reference model using built-in algorithm “best-fit alignment” [30, 31]. Finally, each target model was compared to the reference model to figure out the deviations. The volume, scaling factor, maximum, mean, and standard deviation were recorded (see Supplementary [Supplementary-material supplementary-material-1]). The mean standard deviation was 1.02 mm. The transverse plane was parallel to the foot plate of the loading device. The coronal plane was perpendicular to the ground; the sagittal plane was defined as a plane perpendicular to both the transverse and coronal planes. The origin of the coordinate system was selected as the anterior point of lateral part of the trochlea (Figures 2(a) and 2(b)). The *X*, *Y*, and *Z* axes all passed through the origin and were perpendicular to sagittal, coronal, and transverse planes, respectively (Figures 2(c)–2(e)).

### 2.5. Definition of the Points for Measurement

**Figure 3:**
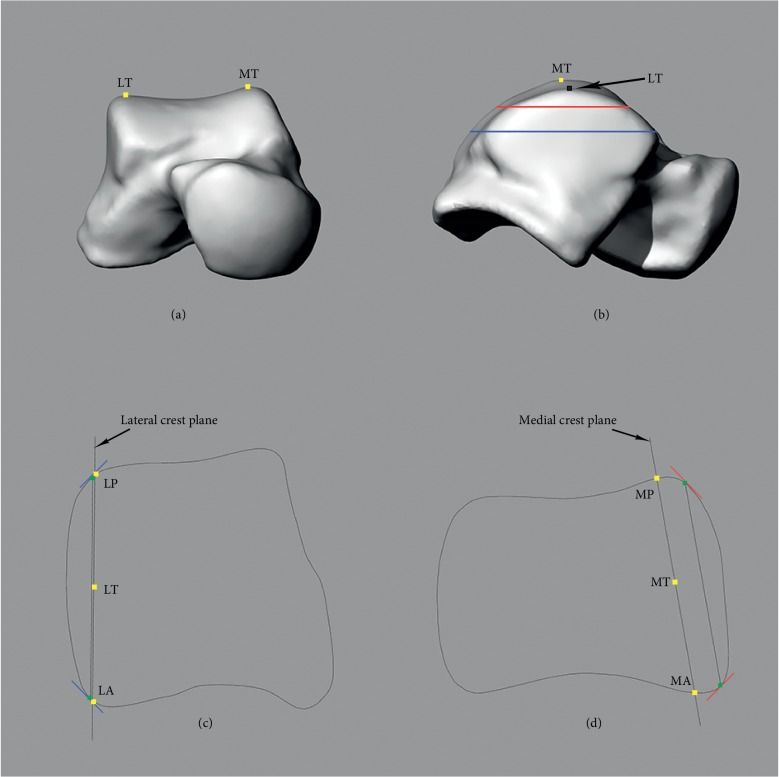
Six points on the circles of lateral and medial crests of the talar dome were defined. (a) LT and MT were defined as the vertex of the lateral and medial crests of the talar dome from front view. (b) Two transverse outlines of the talar dome were obtained including one 8 mm beneath LT (blue line) another 5 mm beneath MT (red line). (c) Two tangent points (green points) were created by two 45-degree lines (blue lines), and the lateral crest plane created two intersections (yellow points) on the transverse outline. (d) Medial crest plane created two intersections (yellow points) on the transverse outline in the same way.

First, lateral-top (LT) and medial-top (MT) points were defined as the vertex of the lateral and medial crests of the talar dome (Figure 3(a)). Second, two transverse outlines on the talar dome, which were 8 mm beneath LT and 5 mm beneath MT, were obtained (Figure 3(b)). Third, two tangent points, which were made by two 45-degree lines on the transverse outline 8 mm beneath LT, were created on the lateral side (Figure 3(c)). Fourth, the lateral crest plane of the talar dome, which passed through LT and was perpendicular to the transverse plane and parallel to the line connecting the two lateral tangent points, was created (Figure 3(c)). Fifth, lateral-anterior (LA) and lateral-posterior (LP) points, which were the two intersections between the lateral crest plane and transverse outline 8 mm beneath LT, were created (Figure 3(c)). Finally, medial-anterior (MA) and medial-posterior (MP) points on the transverse outline 5 mm beneath MT were created in the same way (Figure 3(d)).

### 2.6. Coordinate and Reconstructed Model

Coordinates of the six points for 71 talar models were measured two times by one author (DZ) (see Supplementary [Supplementary-material supplementary-material-1]). Relative distribution of circles on the lateral and medial crests of talar dome included category I, in which *Y* coordinate and *Z* coordinate of LT were both less than MT, category II, in which *Y* coordinate of LT was more than MT and *Z* coordinate of LT was less than MT, category III, in which *Y* coordinate and *Z* coordinate of LT were both more than MT, and category IV, in which *Y* coordinate of LT was less than MT and *Z* coordinate of LT was more than MT.

### 2.7. Radius and Inclination Angle of the Talar Dome

**Figure 4:**
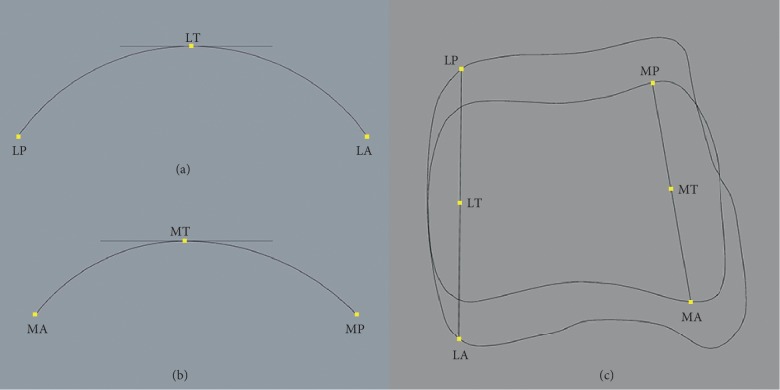
Four parts of circles were defined. (a) A horizontal line which passed through LT was created on the lateral crest plane. Lateral-anterior circle passed through LT and LA and was tangent to the horizontal line. Lateral-posterior circle passed through LT and LP and was tangent to the horizontal line. (b) A horizontal line which passed through MT created the medial crest plane. Medial-anterior circle passed through MT and MA and was tangent to the horizontal line. Medial-posterior circle passed through MT and MP and was tangent to the horizontal line. (c) The inclination angle of the talar dome was the angle between the plane which passed through LT, LA, and LP (lateral crest plane) and the plane which passed through MT, MA, and MP (medial crest plane).

Two horizontal lines on lateral and medial crest planes which passed through LT and MT, respectively, were created (Figures 4(a) and 4(b)). A lateral-anterior circle which passed through LT and LA and tangent to the horizontal line was created on the lateral crest plane. The radius of this circle was defined as the radius of lateral-anterior (R-LA) section of the talar dome. Likewise, the circles and radii of lateral-posterior (R-LP), medial-anterior (R-MA), and medial-posterior (R-MP) sections were defined. Furthermore, the angle between lateral and medial crest planes was defined as inclination angle of the talar dome (Figure 4(c)). Four radii and inclination angles of each model were calculated (see Supplementary [Supplementary-material supplementary-material-1]).

### 2.8. Talocrural Joint Axis and the Orientation

**Figure 5:**
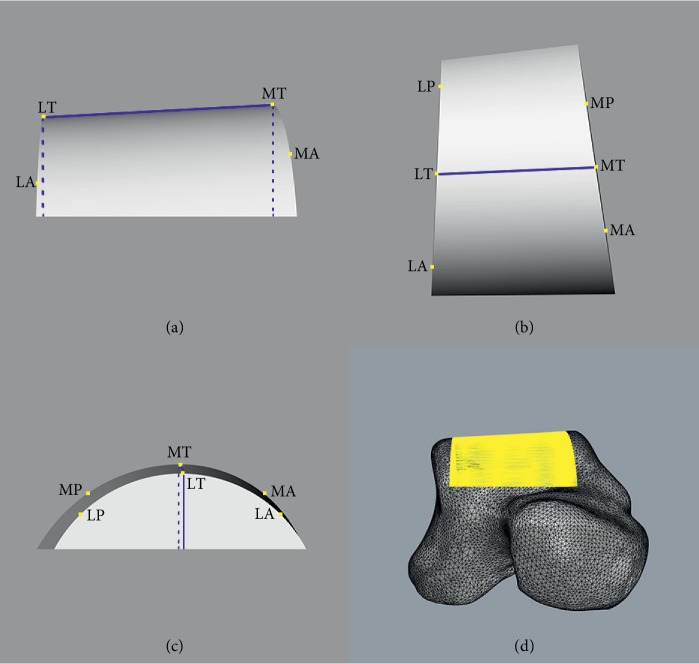
The reconstructed half truncated cone model consisted of two parts with different oriented apexes. (a) The model was observed from front view (blue solid line and dotted line divided the model into two parts). (b) The model was observed from top view (blue solid line divided the model into two parts). (c) The model was observed from lateral view (blue solid line and dotted line divided the model into two parts). (d) The model could be considered as the section between the lateral and medial crest planes of the talar dome (yellow part is the reconstructed half truncated cone model).

A half truncated cone model based on the mean coordinates was reconstructed. And vertical distance from LT point to the bottom transverse plane was 10mm (Figure 5) .This reconstructed model was used for talocrural joint axis analysis. The dorsiflexion axis of the talocrural joint was defined as a line passing through the centers of the lateral-anterior and medial-anterior circles. The plantarflexion axis was defined as a line passing through the centers of the lateral-posterior and medial-posterior circles. The inclination angle was defined as the angle of the talocrural joint axis in the coronal plane, in which a positive value indicated superior oriented axis in the medial direction. The deviation angle was defined as the angle in the transverse plane, in which a positive value indicated anterior oriented axis in the medial direction. The inclination and deviation angles were measured when the reconstructed model was rotated from the neutral position to 30 degrees anterior and 30 degrees posterior with 5 degrees interval. When rotating the model, make sure two points, which included the apexes on the lateral and medial crests of the rotated model, are on the line which connected the mean LT and mean MT point.

### 2.9. Statistical Analysis

A two-tailed unpaired *t*-test was used to investigate gender variations in the inclination angle of the talar dome. A single-factor ANOVA was utilized to identify the differences between R-LA, R-LP, R-MA, and R-MP. When a difference was found, least significant difference (LSD) pairwise multiple comparison tests were applied. SAS software version 9.2 (SAS Institutes, Cary, North Carolina, USA) was used, and a *p* value of < 0.05 was considered significant.

## 3. Results

### 3.1. Relative Distribution of Lateral and Medial Circles

According to the relative distribution category, thirty models belonged to category I, 36 category II, 2 category III, and 3 category IV (Table 1). Mean coordinate of LT, MT, LA, LP, MA, and MP indicated the relative distribution of the lateral and medial circles belonged to category II (Table 2). The reconstructed half truncated cone model, which represented the mean value of the 71 talar models and consisted of two parts of skewed, truncated cones with different oriented apexes, could be considered as the section between the lateral and medial crest planes (Figure 5).

### 3.2. Radius and Inclination Angle of the Talar Dome

**Figure 6:**
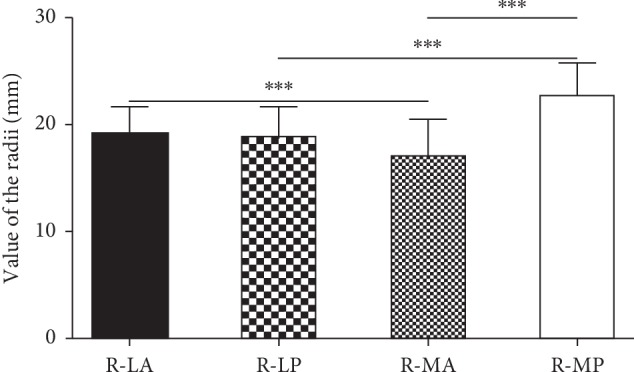
Comparison of the mean value of R-LA with R-LP, R-LA with R-MA, R-LP with R-MP, and R-MA with R-MP (^*∗∗∗*^*p* < 0.001).

The mean values of R-LA, R-LP, R-MA, and R-MP were 19.23 ± 2.47 mm, 18.76 ± 2.90 mm, 17.02 ± 3.49 mm, and 22.75 ± 3.04 mm (Table 3). Significant statistical differences were found between most of the multiple comparisons including R-LA and R-MA, R-LP and R-MP, and R-MA and R-MP (*p* < 0.001); however, no statistical difference (*p*=0.356) was found between R-LA and R-LP (Figure 6). The mean inclination angle of the talar dome was 9.86 ± 3.30 degrees, which was 8.79 ± 2.33 degrees for males and 13.87 ± 3.38 degrees for females, respectively. Gender variation was found in this parameter (*p* < 0.001).

### 3.3. Inclination and Deviation Angles of the Talocrural Joint Axis

**Figure 7:**
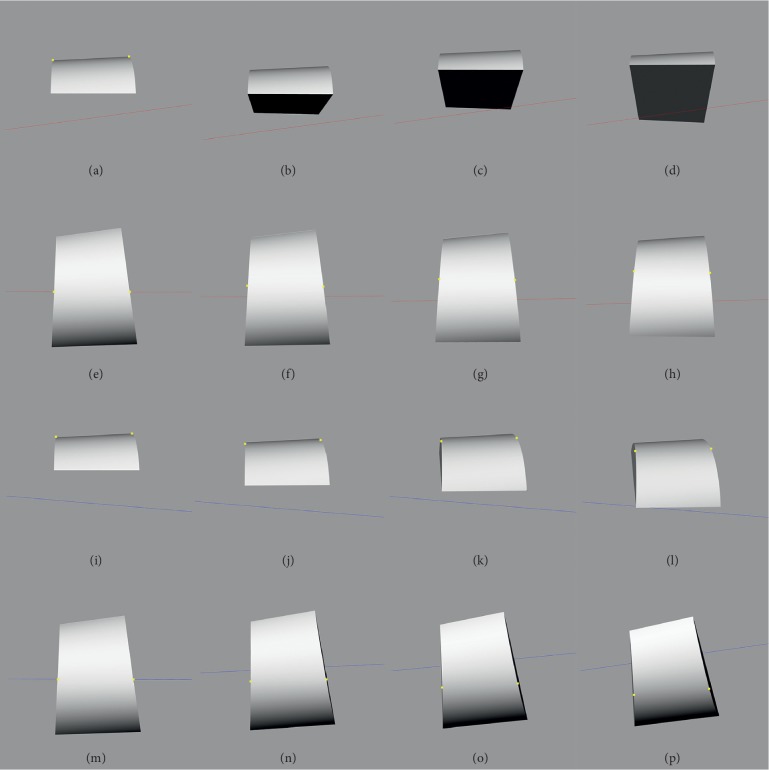
Talocrural joint axis analysis. (a) Inclination angles of the dorsiflexion axis at 0-degree dorsiflexion. (b) Inclination angles of the dorsiflexion axis at 10-degree dorsiflexion. (c) Inclination angles of the dorsiflexion axis at 20-degree dorsiflexion. (d) Inclination angles of the dorsiflexion axis at 30-degree dorsiflexion. (e) Deviation angles of the dorsiflexion axis at 0-degree dorsiflexion. (f) Deviation angles of the dorsiflexion axis at 10-degree dorsiflexion. (g) Deviation angles of the dorsiflexion axis at 20-degree dorsiflexion. (h) Deviation angles of the dorsiflexion axis at 30-degree dorsiflexion. (i) Inclination angles of the plantarflexion axis at 0-degree plantarflexion. (j) Inclination angles of the plantarflexion axis at 10-degree plantarflexion. (k) Inclination angles of the plantarflexion axis at 20-degree plantarflexion. (l) Inclination angles of the plantarflexion axis at 30-degree plantarflexion. (m) Deviation angles of the plantarflexion axis at 0-degree plantarflexion. (n) Deviation angles of the plantarflexion axis at 10-degree plantarflexion. (o) Deviation angles of the plantarflexion axis at 20-degree plantarflexion. (p) Deviation angles of the plantarflexion axis at 30-degree plantarflexion.

The inclination and deviation angles constantly and slightly changed along with the reconstructed model when the reconstructed half truncated cone model was rotated from the neutral position to 30 degrees anterior and 30 degrees posterior (Figure 7). The direction of inclination angle was mainly fixed; however, the direction of the deviation angle was changed during dorsiflexion or plantarflexion, especially for the deviation angle of the plantarflexion axis. The mean inclination and deviation angles were 8.60 ± 0.07 and 0.76 ± 0.69 degrees for the dorsiflexion axis and −7.34 ± 0.07 and 0.09 ± 0.18 degrees for the plantarflexion axis (Table 4).

## 4. Discussion

Better restoration of the morphology of the talar dome is essential to improve the biomechanical research and ankle implant design [11]. However, the results on the radius of the talar dome were controversial, and the repeatability of their methods was not so good [19–22, 24, 26]. The current study found that bilateral asymmetry circles of the medial and lateral crests of the talar dome with four parts of different radii resulted in dorsiflexion and plantarflexion axes constantly changing throughout the talocrural joint motion.

Some limitations existed. First, the neutral position for the foot, ankle, and lower extremity of reference participant was set by the landmark on the skin, which might result in some errors. With the radiographic assessment, it was thought the foot and ankle of the reference participant could be set in neutral position and aligned with ipsilateral lower extremity during CT image collection. Second, rotating the model in this study could not simulate the real talocrural joint motion because the talar simultaneously rotates and slides in the ankle mortise during dorsiflexion and plantarflexion, which were determined and stabilized by the morphology of talar dome and tibial platfond as well as surrounding ligament complexes [16, 32]. It was thought that the simplified motion could not affect the main direction of the talocrural joint axis.

Although 4 categories of the LT and MT relative distribution existed, MT of most models was higher than LT (66/71). The mean *Y* coordinate of LT and MT was nearly the same (mean *Y* coordinate of MT was 0.06 mm less than LT), instead the lateral circle was about 1mm below the medial circle. This indicated the talar dome was saddle-shaped skewed truncated cone with laterally oriented apex which should be copied in ankle prosthetic design. However, a published study indicated the lateral vertex was slightly higher than the medial vertex [26]. The reason for the contradictory findings might be a result of different materials and methods used in our research. They fixed cadaver specimens in the neutral position by bone cement but could not conduct radiographic alignment of the foot and ankle with low extremity [26]. The stress sensor could confirm that all of the first and fifth metatarsal head and calcaneal tuberosity of the reference participant contact with the foot plate during CT scanning. Moreover, the mean standard deviation after model scaling and alignment was similar to that in former studies [30, 31].

Asymmetric talar component of current ankle systems had two different radii on bicondylar that the medial radius was smaller than the lateral one [7]. However, our results indicated that the medial talar dome had two different radii, whereas the lateral had approximately one, and the radius of the medial-posterior part was greater than the lateral radius, whereas the radius of the medial-anterior part was smaller than the lateral radius. This feature may help to improve the current implant design, which might be more anatomical. In this study, four circles were all defined by two points and a tangent line, respectively, which was different from former research studies in which circles were defined by at least three manually created points on lateral or medial crests of the talar dome [11, 22, 25, 26, 33, 34]. It was found that circle defined by three points might result in the apex of the defined circle overtop the actual apex of the talar dome and laed the calculated radius bigger than the actual radius of the talar dome. The volume of the models measured in the study conducted by Nozaki et al. was not available, so we could not compare our results with theirs directly [22]. One study by Nozaki et al. also investigated four radii of the talar dome [22]. The R-LA, R-LP, and R-MP from our study were smaller and R-MA was bigger than those from their results. This difference might result from the medial-anterior part of talus that often presents substantial osteophytes and possibly biases the true dimensions of the talar dome. However, the volume of the models measured in their study was not available, which made it impossible for us to compare the results directly. Therefore, transverse outlines 5 mm rather than 8 mm beneath MT were selected in our research. Furthermore, gender variation was found in the inclination angle of the talar dome. This proved our previous results from the 2D cadaveric study, and it was consistent with a 3D result in which the inclination angle between medial and lateral crest lines was greater in females [26, 35]. It was believed that gender variations in the geometry existed in most bones. Similarly, the anterior-posterior to medial-lateral aspect ratio of distal femoral morphology was larger in women, which indicated that a gender-specific knee implant would reduce the potential for medial-lateral overhang [36]. Based on our results, a gender-specific shape of the talar component might be needed to reduce the potential incompatibility between the shape of the talar component and the bone cutting surface.

It was suggested that two different talocrural joint axes including dorsiflexion and plantarflexion existed. The reconstructed half truncated cone model consisted of two parts with different oriented apexes, which resulted from different radii of circles on the lateral and medial crests as well as their relative distribution. It was demonstrated that the axis of rotation for the talocrural joint slightly changed throughout the motion, which mainly inclined upwards medially during plantarflexion and upwards laterally during dorsiflexion, and the projections on the transverse plane were roughly parallel to the *X* axis. These findings were consistent with the results from the published in vivo studies [3, 16]. Consequently, the ankle implant with these asymmetric features on lateral and medial crests of the talar dome designed by us might better restore the ankle kinematics. However, the inclination and deviation angles of dorsiflexion and plantarflexion axes during neutral position from our research were different from the findings in a recently published study [22]. The potential reason for the variations might due to different research methods. Anterior and posterior circles of lateral or medial crests of the talar dome were tangent in our study. However, the two circles of lateral or medial crest defined in their study were separated [22].

It was found that medial crests of the talar dome consisted of two circles with different radii, of which the posterior radius was bigger, and the radius of lateral-anterior crests was nearly the same as the lateral-posterior part. The lateral radius was bigger than the medial-anterior radius but smaller than medial-posterior radius. In addition, the vertex of the medial crest was slightly higher than the lateral crest. Moreover, it is suggested these features resulted in that the dorsiflexion and plantarflexion axes were different and both slightly changed throughout the ankle motion, and the ankle kinematics might be close to physiological state. However, no talar component of the current ankle systems copied these features, which should be considered in future biomechanical and clinical research such as design of ankle implant.

## Figures and Tables

**Table 1:** Relative distribution of lateral and medial circles.

	*Y* coordinate	*Z* coordinate	Number of models
Category I	LT > MT	LT < MT	30
Category II	LT < MT	LT < MT	36
Category III	LT < MT	LT > MT	2
Category IV	LT > MT	LT > MT	3

LT: lateral-top point; MT: medial-top point.

**Table 2:** Mean coordinate of LT, MT, LA, LP, MA, and MP.

	*X* coordinate (mm)	*Y* coordinate (mm)	*Z* coordinate (mm)
LT	−5.23	16.94	9.89
LA	−5.83	1.40	1.89
LP	−4.65	32.21	1.89
MT	17.59	16.88	10.98
MA	19.18	5.03	5.98
MP	15.70	30.93	5.98

LT: lateral-top point; MT: medial-top point; LA: lateral-anterior point; LP: lateral-posterior point; MA: medial-anterior point; MP: medial-posterior point.

**Table 3:** Values of R-LA, R-LP, R-MA, and R-MP.

Variable	Value (mm)
R-LA	19.23 ± 2.47
R-LP	18.76 ± 2.90
R-MA	17.02 ± 3.49
R-MP	22.75 ± 3.04

Values represent means ± standard deviation; LT: lateral-top point; MT: medial-top point; LA: lateral-anterior point; LP: lateral-posterior point; MA: medial-anterior point; MP: medial-posterior point.

**Table 4:** Inclination and deviation angles of the dorsiflexion and plantarflexion axes.

Dorsiflexion or plantarflexion (degree)	Dorsiflexion axis		Plantarflexion axis	
	Inclination angle (degree)	Deviation angle (degree)	Inclination angle (degree)	Deviation angle (degree)
0	8.49	−0.15	−7.24	−0.15
5	8.53	0.13	−7.28	−0.08
10	8.57	0.41	−7.31	−0.01
15	8.61	0.71	−7.34	0.07
20	8.64	1.04	−7.37	0.15
25	8.67	1.39	−7.40	0.25
30	8.69	1.78	−7.43	0.37
Mean values ± SD	8.60 ± 0.07	0.76 ± 0.69	−7.34 ± 0.07	0.09 ± 0.18

SD: standard deviation.

## Data Availability

The datasets supporting the conclusions of this article are included within the article and in the supplementary materials.

## Supplementary Materials

Supplementary Materials The supplementary materials of the present study included three supplementary data files, which are referred in the manuscript and are all the raw data of this original research.
