# Supplementary material for: Talar Dome Investigation and Talocrural Joint Axis Analysis Based on Three-Dimensional (3D) Models: Implications for Prosthetic Design
Source: Biomed Res Int. 2019 Nov 7;2019:8634159. doi: 10.1155/2019/8634159 (PMC6885182; doi:10.1155/2019/8634159)
Supplement: Supplementary Materials — The supplementary materials of the present study included three supplementary data files, which are referred in the manuscript and are all the raw data of this original research. [file 8634159.f1.zip › 8634159.f1/Supplementary data 1.pdf]

ID	Gender	Age	Side	Volume (mm^3)	Scaling Factor	Theoretical volume after scaling (mm^3)	Actual volume after scaling (mm^3)	Maximum deviation (+/-mm)	Mean deviation (+/-mm)	Standard deviation (+/-mm)
Reference Model	Male	33	Right	35741.925	——	——	——	——	——	——
Model 1	Male	23	Right	20783.265	1.198086169	35741.925	35741.9253	3.157892/-3.593347	0.770149/-0.695618	0.928674
Model 2	Male	23	Left-Right	41595.1025	0.950703837	35741.925	35741.925	2.262826/-3.183660	0.593617/-0.643036	0.776396
Model 3	Female	24	Right	20734.5793	1.199023156	35741.925	35739.85431	3.379233/-5.097486	0.785568/-0.713110	0.95337
Model 4	Female	24	Left-Right	41770.6157	0.949370401	35741.925	35741.925	18.552594/-13.790482	0.768520/-0.758705	0.969156
Model 5	Female	23	Right	28560.8903	1.077627666	35741.925	35741.9249	3.435085/-5.588277	0.954041/-0.977117	1.235117
Model 6	Female	23	Left-Right	37462.4503	0.984450581	35741.925	35741.925	6.763486/-14.522540	0.853092/-0.847816	1.044702
Model 7	Male	25	Right	25103.9477	1.124981306	35741.925	35741.925	2.617108/-2.728633	0.728027/-0.682917	0.86668
Model 8	Male	25	Left-Right	36853.0049	0.989847624	35741.925	35741.9251	2.337259/-3.687091	0.614734/-0.638816	0.793677
Model 9	Male	24	Right	40792.8078	0.95689609	35741.925	35741.925	3.305263/-2.953718	0.706351/-0.790509	0.92776
Model 10	Male	24	Left-Right	38304.7311	0.977181359	35741.925	35741.925	6.423891/-5.185723	1.023797/-0.898173	1.203316
Model 11	Female	24	Right	40026.7219	0.962962345	35741.925	35741.925	4.242726/-3.800814	0.763882/-0.855414	1.030981
Model 12	Female	24	Left-Right	24990.7754	1.126676932	35741.925	35741.9253	3.801853/-17.152144	0.608962/-0.729055	0.874807
Model 13	Female	25	Right	40896.1634	0.956089299	35741.925	35741.925	2.601776/-3.162335	0.695054/-0.772926	0.904851
Model 14	Female	25	Left-Right	33713.2353	1.01966889	35741.925	35741.9249	5.146033/-5.292232	0.893663/-0.911051	1.113735
Model 15	Male	25	Right	32865.77	1.028358896	35741.925	35741.9248	4.129822/-4.479134	0.841462/-0.936646	1.171206
Model 16	Male	25	Left-Right	23394.7302	1.151736948	35741.925	35741.925	3.344808/-3.465082	0.695311/-0.764837	0.906114
Model 17	Male	23	Right	40391.67	0.96005337	35741.925	35741.9249	4.360391/-4.527151	0.847608/-0.811796	1.072738
Model 18	Male	23	Left-Right	38018.8075	0.979624902	35741.925	35741.925	19.537212/-26.177775	1.066136/-0.947070	1.319956
Model 19	Male	29	Left-Right	32921.3235	1.027780131	35741.925	35741.925	3.026570/-3.248101	0.665256/-0.701632	0.833231
Model 20	Male	25	Right	28589.6908	1.077265687	35741.925	35745.34044	3.944387/-5.590017	1.007763/-0.880149	1.189092
Model 21	Male	25	Left-Right	36411.733	0.993830216	35741.925	35741.9251	5.344665/-6.311435	0.822739/-0.730789	0.967686
Model 22	Male	26	Right	31826.5268	1.039432354	35741.925	35741.9251	3.844747/-14.522124	0.780008/-0.706997	0.967794
Model 23	Male	26	Left-Right	35543.9176	1.00185349	35741.925	35741.9251	2.661962/-3.256108	0.558603/-0.623196	0.746992
Model 24	Male	24	Right	24172.3029	1.139252448	35741.925	35741.925	3.373058/-5.164954	0.890671/-0.765131	1.028246
Model 25	Male	24	Left-Right	33887.9813	1.017913201	35741.925	35741.9252	3.245365/-7.354352	0.724923/-0.912750	1.010438
Model 26	Male	26	Left-Right	44085.6833	0.93245265	35741.925	35741.925	4.062068/-9.758494	0.897768/-0.853881	1.075635
Model 27	Male	23	Right	35425.8988	1.00296479	35741.925	35741.925	3.597347/-5.516878	0.850293/-0.853607	1.04815
Model 28	Male	23	Left-Right	33149.3806	1.025417773	35741.925	35741.9249	2.985810/-3.538595	0.787531/-0.930019	1.019327
Model 29	Male	23	Left-Right	37631.7239	0.98297229	35741.925	35741.925	4.555446/-9.355408	0.703824/-0.811884	0.969223
Model 30	Male	37	Right	31904.77	1.038581958	35741.925	35741.9251	3.178309/-2.712099	0.602438/-0.643377	0.780016
Model 31	Male	37	Left-Right	27797.0147	1.087409845	35741.925	35741.9246	2.781580/-3.304051	0.645179/-0.726207	0.841461
Model 32	Male	25	Right	32422.8738	1.033020193	35741.925	35741.9251	3.631003/-4.133767	0.914468/-0.823377	1.09496
Model 33	Male	25	Left-Right	31767.9667	1.040070648	35741.925	35741.925	4.190417/-3.730994	0.982749/-0.827970	1.176206
Model 34	Male	24	Right	31245.1395	1.045839781	35741.925	35741.925	3.639292/-4.186858	0.989649/-1.001199	1.246855
Model 35	Male	24	Left-Right	27864.7847	1.086527566	35741.925	35741.9251	3.373040/-6.285696	0.929236/-0.849548	1.132083
Model 36	Male	24	Right	34305.2305	1.013769449	35741.925	35741.8775	3.171711/-3.431430	0.734287/-0.762073	0.932505
Model 37	Male	24	Left-Right	27797.0147	1.087409845	35741.925	35741.9258	2.6634.4/-3.096111	0.678908/-0.723907	0.869367
Model 38	Female	21	Right	34616.9785	1.010717054	35741.925	35741.925	4.950884/-5.887907	1.256615/-1.014109	1.40859
Model 39	Female	21	Left-Right	28216.9633	1.081988277	35741.925	35741.925	3.642392/-5.436542	0.938994/-0.845298	1.171632
Model 40	Male	25	Right	42158.2685	0.94645156	35741.925	35741.925	18.567637/-12.563596	0.631426/-0.760820	0.914301
Model 41	Male	25	Left-Right	28595.3793	1.077194248	35741.925	35741.9248	5.242800/-5.442852	1.048483/-0.789774	1.20742
Model 42	Female	25	Right	24412.8366	1.1354985	35741.925	35741.925	2.653462/-3.594617	0.773417/-0.743792	0.946653
Model 43	Female	25	Left-Right	35934.0525	0.998214591	35741.925	35741.925	19.393084/-20.838455	1.003462/-0.954733	1.2333074
Model 44	Male	22	Right	20749.8346	1.198729243	35741.925	35741.9251	4.194742/-3.708754	0.818261/-0.789496	1.008722
Model 45	Male	22	Left-Right	26310.3025	1.10751776	35741.925	35741.9252	3.841344/-3.532820	0.598665/-0.791373	0.859825
Model 46	Male	23	Right	36362.8052	0.994275764	35741.925	35741.9251	9.283103/-10.373791	1.117650/-0.934958	1.338725
Model 47	Male	23	Left-Right	37660.233	0.982724188	35741.925	35741.9251	7.037375/-14.885823	0.958793/-0.913222	1.198659
Model 48	Female	23	Right	24680.4597	1.131379308	35741.925	35741.8958	3.014781/-3.468129	0.568320/-0.709896	0.81476
Model 49	Female	23	Left-Right	34358.2614	1.013247607	35741.925	35736.88736	2.403005/-2.982314	0.773111/-0.935299	1.009593
Model 50	Male	27	Right	27712.1639	1.088518547	35741.925	35741.9254	2.696040/-3.374178	0.712895/-0.701963	0.888824
Model 51	Male	27	Left-Right	33380.9675	1.023040923	35741.925	35741.925	3.767453/-4.388392	0.777805/-0.747546	0.940798
Model 52	Male	23	Right	39507.4253	0.967163141	35741.925	35741.925	4.285828/-8.023424	0.792026/-0.843689	1.01699
Model 53	Male	23	Left-Right	36663.7331	0.991548021	35741.925	35741.9249	2.811137/-2.724569	0.543644/-0.612953	0.744413
Model 54	Male	31	Right	34800.8235	1.008934113	35741.925	35741.913	4.136646/-4.920510	0.731107/-0.866708	0.996422
Model 55	Male	31	Left-Right	32096.8188	1.036506388	35741.925	35741.9251	2.769191/-2.628649	0.622540/-0.766429	0.843552
Model 56	Male	26	Right	34796.563	1.008975289	35741.925	35741.9249	3.237077/-2.774594	0.657659/-0.643194	0.800744
Model 57	Male	26	Left-Right	36525.4648	0.992797624	35741.925	35741.925	8.347384/-14.063855	1.084861/-1.100679	1.427113
Model 58	Male	26	Right	34860.0742	1.00836217	35741.925	35735.7794	8.811606/-14.179803	1.311248/-0.961936	1.507541
Model 59	Male	26	Left-Right	37822.4473	0.98131726	35741.925	35741.925	9.616632/-6.160644	0.862132/-0.845070	1.100568
Model 60	Male	33	Left-Right	42948.7834	0.940608771	35741.925	35741.925	2.813520/-2.909138	0.600337/-0.650196	0.808028
Model 61	Male	25	Right	27257.5349	1.094537018	35741.925	35738.29872	3.143398/-3.242590	0.739829/-0.700021	0.913241
Model 62	Male	25	Left-Right	28327.5271	1.080578756	35741.925	35741.925	2.503066/-3.518590	0.684467/-0.627850	0.842021
Model 63	Male	25	Right	45896.6953	0.920023331	35741.925	35741.9249	13.681707/-15.807611	0.828722/-0.790227	1.029157
Model 64	Female	22	Left-Right	36230.3765	0.995485711	35741.925	35741.9248	7.443108/-10.651171	1.102204/-1.118578	1.425297
Model 65	Male	27	Right	25476.0809	1.119476823	35741.925	35741.925	3.240059/-3.782717	0.554158/-0.627727	0.758642
Model 66	Male	27	Left-Right	28728.4885	1.075527999	35741.925	35741.925	3.485491/-4.297899	0.872956/-0.818389	1.04927
Model 67	Male	26	Right	30806.1943	1.050783625	35741.925	35743.59596	4.498279/9.416327	0.847528/-0.707463	1.010976
Model 68	Male	26	Left-Right	35687.8989	1.000504362	35741.925	35741.925	20.252418/-19.986516	1.103086/-1.019368	1.389093
Model 69	Male	24	Right	30321.3984	1.056354218	35741.925	35741.9248	4.021187/-5.213835	0.792559/-0.824457	1.061079
Model 70	Male	24	Left-Right	36829.5516	0.990057693	35741.925	35741.9249	4.441529/-24.466465	0.742161/-0.830260	0.971045
