# Supplementary material for: Talar Dome Investigation and Talocrural Joint Axis Analysis Based on Three-Dimensional (3D) Models: Implications for Prosthetic Design
Source: Biomed Res Int. 2019 Nov 7;2019:8634159. doi: 10.1155/2019/8634159 (PMC6885182; doi:10.1155/2019/8634159)
Supplement: Supplementary Materials — The supplementary materials of the present study included three supplementary data files, which are referred in the manuscript and are all the raw data of this original research. [file 8634159.f1.zip › 8634159.f1/Supplementary data 2.pdf]

ID	Gender	Age	Side	Point	X (mm)	Y (mm)	Z (mm)
Reference Model	Male	33	Right	LT	-3.663398743	17.75759888	8.921703339
				LA	-3.91869162	0.567282063	0.921703339
				LP	-3.435537655	33.10077776	0.921703339
				MT	18.89550018	17.56370544	11.29190063
				MA	20.58946185	4.721359594	6.291900635
				MP	17.03645279	30.94700423	6.291900635
Model 1	Male	23	Right	LT	-5.852046967	16.90316772	10.40742493
				LA	-6.34910385	2.313868422	2.407424927
				LP	-5.33524576	32.0719899	2.407424927
				MT	17.83791733	17.96780396	11.73705292
				MA	19.05205781	5.765507093	6.737052917
				MP	16.46302432	31.78568819	6.737052917
Model 2	Male	23	Left-Right	LT	-4.651634216	17.99337769	10.36100006
				LA	-5.753331193	1.843007873	2.361000061
				LP	-3.703264119	32.23439145	2.361000061
				MT	18.085186	17.26167297	12.05571365
				MA	19.73555436	4.953273466	7.055713654
				MP	16.26475033	30.83842966	7.055713654
Model 3	Female	24	Right	LT	-6.004547119	16.93792725	9.899082184
				LA	-7.732145679	2.17970787	1.899082184
				LP	-4.169406454	32.61483805	1.899082184
				MT	18.09893799	14.05014038	11.32800293
				MA	19.56072218	4.776227636	6.32800293
				MP	15.6049949	29.87231903	6.32800293
Model 4	Female	24	Left-Right	LT	-1.865398407	16.84620667	9.464179993
				LA	-2.947103804	0.819889124	1.464179993
				LP	-0.594608541	35.67897862	1.464179993
				MT	16.97156525	17.75267029	11.0717659
				MA	18.02476421	5.052184436	6.0717659
				MP	15.80915685	31.77010854	6.0717659
Model 5	Female	23	Right	LT	-8.304473877	17.06719971	10.10242081
				LA	-9.476300566	4.402004637	2.102420807
				LP	-7.057154645	30.54832413	2.102420807
				MT	17.71366501	16.59442139	11.33443069
				MA	20.28927721	4.941821504	6.334430695
				MP	14.52501436	31.02053345	6.334430695
Model 6	Female	23	Left-Right	LT	-7.215572357	17.29013062	9.966884613
				LA	-8.54751941	2.587426802	1.966884613
				LP	-6.002941378	30.67576339	1.966884613
				MT	18.32833481	18.13085938	11.32507324
				MA	19.79082733	6.063875231	6.325073242
				MP	16.71621122	31.43244518	6.325073242
Model 7	Male	25	Right	LT	-5.074958801	16.19920349	10.4234848
				LA	-5.617127547	0.296791656	2.423484802
				LP	-4.524615554	32.34138255	2.423484802
				MT	17.56097794	15.58891296	11.11849213
				MA	19.19679761	5.120066	6.118492126
				MP	15.3614182	29.66555883	6.118492126
Model 8	Male	25	Left-Right	LT	-6.193031311	17.44224548	10.07034683
				LA	-7.386234054	2.794605677	2.070346832
				LP	-5.029059225	31.73105261	2.070346832
				MT	16.92798615	14.77351379	10.94964981
				MA	18.32977564	4.485673189	5.949649811
				MP	14.7987017	30.40049566	5.949649811
Model 9	Male	24	Right	LT	-3.592140198	16.59147644	9.55979538
				LA	-3.985202111	-0.407576234	1.55979538
				LP	-3.190527935	33.96031269	1.55979538
				MT	14.56023026	15.82987976	11.10790253
				MA	16.70020182	3.2009816	6.107902527
				MP	11.93960036	31.29535071	6.107902527
Model 10	Male	24	Left-Right	LT	-5.243247986	16.12339783	9.49426651
				LA	-4.24551446	-7.67E-02	1.49426651
				LP	-6.237485735	32.26673622	1.49426651
				MT	15.42416382	16.66091919	10.72824478
				MA	17.91754731	3.793543338	5.728244781
				MP	12.61631683	31.15111803	5.728244781
Model 11	Female	24	Right	LT	-4.784915924	14.21134949	9.887439728
				LA	-5.063418284	0.602438929	1.887439728
				LP	-4.418084043	32.13644807	1.887439728
				MT	18.25575638	16.87013245	11.43972397
				MA	20.07492644	5.379809714	6.439723969
				MP	15.84194219	32.1163728	6.439723969

Model 12	Female	24	Left-Right	LT	-5.342258453	16.06195068	9.52481842
				LA	-6.366970943	1.782648422	1.52481842
				LP	-4.301092034	30.98154845	1.52481842
				MT	16.44877625	19.17475891	10.63125229
				MA	20.07665696	4.029755242	5.631252289
				MP	13.32916151	32.00734496	5.631252289
Model 13	Female	25	Right	LT	-5.693172455	16.65444946	9.886730194
				LA	-6.352010616	1.726849662	1.886730194
				LP	-5.033597791	31.52064534	1.886730194
				MT	17.24755096	16.7437439	11.40744781
				MA	19.33935594	6.28856483	6.407447815
				MP	14.49800402	30.55894619	6.407447815
Model 14	Female	25	Left-Right	LT	-7.240936279	16.20176697	10.13902664
				LA	-8.433655723	2.864631579	2.139026642
				LP	-5.862174167	31.6067739	2.139026642
				MT	18.22592545	16.95994568	11.17168808
				MA	20.78803592	4.163015365	6.17168808
				MP	15.52473628	30.48974202	6.17168808
Model 15	Male	25	Right	LT	-5.569812775	18.05827332	10.37462997
				LA	-6.11003074	2.016287828	2.374629974
				LP	-5.183372789	32.08542284	2.374629974
				MT	16.88996887	16.79940796	11.12582779
				MA	18.31643844	5.219954627	6.125827789
				MP	15.03497366	31.53477796	6.125827789
Model 16	Male	25	Left-Right	LT	-5.575267792	17.46894836	10.43527985
				LA	-6.50152684	2.913994973	2.435279846
				LP	-4.605617552	32.70573776	2.435279846
				MT	16.94054031	17.00698853	11.22231293
				MA	18.02372924	5.721816934	6.222312927
				MP	15.60236923	30.94868413	6.222312927
Model 17	Male	23	Right	LT	-3.390026093	17.66897583	9.543117523
				LA	-3.193416767	0.316568962	1.543117523
				LP	-3.563006537	32.93593757	1.543117523
				MT	19.65153885	14.76940918	10.45146179
				MA	20.74007664	3.993434703	5.451461792
				MP	18.15177033	29.6163602	5.451461792
Model 18	Male	23	Left-Right	LT	-3.905368805	16.579422	9.484302521
				LA	-4.630746396	0.522909148	1.484302521
				LP	-3.152624487	33.22936377	1.484302521
				MT	19.85984802	15.10870361	10.71500015
				MA	21.33082759	4.529247293	5.715000153
				MP	17.92848174	29.10765198	5.715000153
Model 19	Male	29	Left-Right	LT	-6.267341614	15.67240906	9.525867462
				LA	-7.257781907	1.556488706	1.525867825
				LP	-5.817366907	31.94296871	1.525867825
				MT	16.77455521	17.60134888	10.19289017
				MA	17.58050073	5.722674044	5.192890167
				MP	15.72026813	31.3357516	5.192890167
Model 20	Male	25	Right	LT	-7.043258667	15.7822113	10.73516846
				LA	-7.18974729	2.544376149	2.735168457
				LP	-6.871429104	31.31004811	2.735168457
				MT	17.11155701	19.07023621	11.8181839
				MA	19.14555365	7.745004218	6.818183899
				MP	14.68860696	32.56114897	6.818183899
Model 21	Male	25	Left-Right	LT	-8.159618378	15.98512268	10.05912018
				LA	-7.540961691	2.764038872	2.059120178
				LP	-8.720753547	27.97693536	2.059120178
				MT	17.52416611	17.53364563	11.93920517
				MA	19.29270512	6.664708365	6.93920517
				MP	15.17496554	31.97116352	6.93920517
Model 22	Male	26	Right	LT	-5.963924408	17.39108276	9.690567017
				LA	-6.103905047	0.802514802	1.690567017
				LP	-5.837107492	32.41966847	1.690567017
				MT	17.53948212	18.01831055	11.33818054
				MA	18.62073457	5.63644153	6.338180542
				MP	16.27506165	32.4977123	6.338180542
Model 23	Male	26	Left-Right	LT	-3.313262939	17.11216736	9.585037231
				LA	-4.13645295	0.505108827	1.585037231
				LP	-2.451104023	34.48850182	1.585037231
				MT	18.79735565	17.59887695	11.29112625
				MA	19.54473272	4.686114508	6.291126251
				MP	17.91123653	32.15945182	6.291126251

Model 24	Male	24	Right	LT	-5.792190552	16.27690125	11.26179504
				LA	-6.383432699	1.430683033	3.261795044
				LP	-5.171438094	32.2938696	3.261795044
				MT	16.8089447	16.9052124	11.50444031
				MA	18.09045568	7.363423466	6.504440308
				MP	15.11895091	29.23875645	6.504440308
Model 25	Male	24	Left-Right	LT	-5.574668884	17.29414368	10.9743576
				LA	-5.683574887	1.541100184	2.974357605
				LP	-5.470030357	32.42990504	2.974357605
				MT	17.10151672	17.2747345	12.23970413
				MA	18.56944424	7.424974228	7.239704132
				MP	15.20342015	30.01091931	7.239704132
Model 26	Male	26	Left-Right	LT	-6.037700653	16.58886719	8.996425629
				LA	-5.726197168	0.254711559	0.996425629
				LP	-6.387631121	33.91238134	0.996425629
				MT	18.8064537	17.1190033	9.438995361
				MA	20.41251937	5.497523354	4.438995361
				MP	16.75673675	31.9507408	4.438995361
Model 27	Male	23	Right	LT	-6.520523071	16.40336609	9.452022552
				LA	-6.994660615	0.757987612	1.452022552
				LP	-6.008236969	33.31568178	1.452022552
				MT	18.15511703	18.40228271	10.33620071
				MA	18.80242242	4.133356446	5.336200714
				MP	17.52715024	32.25417393	5.336200714
Model 28	Male	23	Left-Right	LT	-6.107318878	17.69273376	9.585609436
				LA	-6.574459989	0.252705782	1.585609436
				LP	-5.697682366	32.98591192	1.585609436
				MT	17.67356491	19.01907349	10.59771347
				MA	18.289551	4.205595259	5.59771347
				MP	17.12295099	32.26045568	5.59771347
Model 29	Male	23	Left-Right	LT	-2.17432785	19.59571838	9.897323608
				LA	-3.214135055	2.446422567	1.897323608
				LP	-1.201208857	36.34409975	1.897323608
				MT	17.92238998	17.68904114	10.60002899
				MA	19.79434172	5.944099941	5.600028992
				MP	15.75115464	31.28749726	5.600028992
Model 30	Male	37	Right	LT	-6.832103729	16.97807312	10.07195663
				LA	-7.809605555	1.853280491	2.071956635
				LP	-5.950187165	30.6238841	2.071956635
				MT	19.31577682	16.60444641	11.59056091
				MA	20.31273303	5.650615258	6.590560913
				MP	18.06589035	30.33729182	6.590560913
Model 31	Male	37	Left-Right	LT	-5.093547821	17.61045837	9.003475189
				LA	-4.948878826	0.331490129	1.003475189
				LP	-5.148532965	33.29138344	1.003475189
				MT	18.4468956	17.29801941	9.977443695
				MA	20.10529231	5.976669873	4.977443695
				MP	16.18093984	32.61902361	4.977443695
Model 32	Male	25	Right	LT	-4.888912201	17.40953064	9.721343994
				LA	-4.813442485	0.318228237	1.721343994
				LP	-4.954196037	32.19408063	1.721343994
				MT	17.4920578	18.21711731	11.67586899
				MA	19.37281429	5.677079696	6.675868988
				MP	15.43291759	31.94653727	6.675868988
Model 33	Male	25	Left-Right	LT	-6.001785278	16.1174469	9.705863953
				LA	-6.514888435	1.632785164	1.705863953
				LP	-5.477007025	31.12773601	1.705863953
				MT	17.29774094	18.07397461	10.83683014
				MA	18.97494977	4.339851445	5.836830139
				MP	15.66829239	31.4258903	5.836830139
Model 34	Male	24	Right	LT	-5.813434601	18.0940094	10.65917206
				LA	-7.306118738	2.932676175	2.659172058
				LP	-4.425152987	32.19491637	2.659172058
				MT	18.06517029	16.08314514	11.67885971
				MA	19.49893956	4.401439108	6.678859711
				MP	16.4396281	29.32733049	6.678859711
Model 35	Male	24	Left-Right	LT	-5.78830719	19.07391357	10.78757477
				LA	-7.602941876	3.150868828	2.787574768
				LP	-4.307368351	32.17163595	2.787574768
				MT	18.7431488	14.5269165	11.33007813
				MA	19.65923885	4.249514906	6.330078125
				MP	17.53040469	27.99284338	6.330078125

Model 36	Male	24	Right	LT	-6.593544006	16.9936676	9.894943237
				LA	-7.588558782	1.711944479	1.894943237
				LP	-5.641524417	31.69232638	1.894943237
				MT	20.08459473	15.84411621	9.989334106
				MA	21.09840217	5.190898248	4.989334106
				MP	18.82509217	29.0078571	4.989334106
Model 37	Male	24	Left-Right	LT	-6.136257172	18.4155426	9.617183685
				LA	-7.690933991	1.716981171	1.617183685
				LP	-4.781320991	33.01179253	1.617183685
				MT	18.93540955	15.6656189	10.02190399
				MA	20.40417735	4.687331299	5.021903992
				MP	17.02866115	29.86362208	5.021903992
Model 38	Female	21	Right	LT	-3.348747253	15.72407532	9.918773651
				LA	-3.850123221	0.925779699	1.918773651
				LP	-2.825001073	31.71457326	1.918773651
				MT	16.33501816	17.59179688	12.02871323
				MA	17.95346074	5.944729547	7.028713226
				MP	14.71504545	29.27816276	7.028713226
Model 39	Female	21	Left-Right	LT	-5.226486206	17.25886536	10.24526978
				LA	-6.163873067	1.823864507	2.245269775
				LP	-4.326208292	32.08283073	2.245269775
				MT	17.19179535	14.81565857	11.78133011
				MA	19.10682696	4.82166787	6.781330109
				MP	14.29794289	29.91782947	6.781330109
Model 40	Male	25	Right	LT	-3.660293579	17.87721252	10.14382172
				LA	-3.560301071	1.934004519	2.143821716
				LP	-3.757645438	33.39938474	2.143821716
				MT	15.80896378	17.86740112	11.94919205
				MA	17.38339344	5.930103813	6.949192047
				MP	14.06671544	31.07709655	6.949192047
Model 41	Male	25	Left-Right	LT	-6.083999634	17.28947449	9.145591736
				LA	-7.891794907	2.369638706	1.145591825
				LP	-4.466934907	31.23619871	1.145591825
				MT	17.51585388	16.02374268	9.998241425
				MA	18.79830713	4.013500086	4.998241425
				MP	15.97019913	30.42033017	4.998241425
Model 42	Female	25	Right	LT	-4.490447998	14.80543518	8.745079041
				LA	-5.45924626	1.423192526	0.745079041
				LP	-3.172154589	32.82672048	0.745079041
				MT	16.35372925	18.17788696	9.966712952
				MA	20.79014118	2.787398895	4.966712952
				MP	12.23508494	32.42381184	4.966712952
Model 43	Female	25	Left-Right	LT	-5.75409317	16.23248291	9.203105927
				LA	-6.477771471	1.628701255	1.203105927
				LP	-5.216463907	31.37757871	1.203105825
				MT	18.43527222	14.56382751	11.08588791
				MA	19.73541065	4.20150842	6.085887909
				MP	16.30162958	31.49353574	6.085887909
Model 44	Male	22	Right	LT	-1.296604156	15.95504761	9.821155548
				LA	-1.555965119	-0.301632608	1.821155548
				LP	-1.030384689	32.64161718	1.821155548
				MT	17.53574371	17.19787598	11.45176697
				MA	19.19198379	5.191697685	6.451766968
				MP	15.80204526	29.76555441	6.451766968
Model 45	Male	22	Left-Right	LT	-1.843460083	15.76011658	9.932666779
				LA	-1.868947744	-0.537479685	1.932666779
				LP	-1.818811601	31.52111596	1.932666779
				MT	17.36813736	15.35673523	11.69410324
				MA	19.30824472	4.199705064	6.694103241
				MP	14.94275846	29.30443017	6.694103241
Model 46	Male	23	Right	LT	-5.499526978	17.94694519	10.24858475
				LA	-5.84102889	0.841713403	2.248584747
				LP	-5.220318457	32.14012046	2.248584747
				MT	18.95137024	16.49266052	10.08184814
				MA	20.64488626	5.909799955	5.081848145
				MP	16.37887823	32.58889296	5.081848145
Model 47	Male	23	Left-Right	LT	-4.687828064	17.79560852	9.74369812
				LA	-5.582969691	1.090449758	1.74369812
				LP	-3.854780456	33.34196693	1.74369812
				MT	17.92629242	15.23049927	10.55042648
				MA	18.87684485	5.49257316	5.550426483
				MP	16.40382477	30.82740589	5.550426483

Model 48	Female	23	Right	LT	-4.41255188	15.94737244	8.812660217
				LA	-4.453982642	1.059753783	0.812660217
				LP	-4.384138686	33.36207001	0.812660217
				MT	16.11403275	18.14305115	10.29188156
				MA	19.62031079	3.671325275	5.291881561
				MP	12.74275902	31.9368278	5.291881561
Model 49	Female	23	Left-Right	LT	-0.870201111	15.42893982	8.886993408
				LA	-0.252794129	0.791200466	0.886993408
				LP	-1.628486729	33.46836547	0.886993408
				MT	15.94839859	17.54890442	10.65337372
				MA	19.93808285	3.463742824	5.653373718
				MP	11.92663658	31.6447096	5.653373718
Model 50	Male	27	Right	LT	-4.753170013	17.47329712	9.617076874
				LA	-4.58410792	0.846061935	1.617076874
				LP	-4.894235994	31.34711826	1.617076874
				MT	17.4376297	17.02928162	9.67357254
				MA	18.72299401	4.938848047	4.67357254
				MP	15.87823805	31.69727968	4.67357254
Model 51	Male	27	Left-Right	LT	-5.847602844	18.60920715	10.45144272
				LA	-6.36750415	1.646919856	2.451442719
				LP	-5.462028546	31.1889448	2.451442719
				MT	15.50682831	18.26582336	10.12742996
				MA	17.03058208	5.149677868	5.127429962
				MP	14.02208287	31.04619355	5.127429962
Model 52	Male	23	Right	LT	-6.710124969	16.89067078	10.43453979
				LA	-7.091369391	2.105429006	2.434539795
				LP	-6.378372944	29.75652173	2.434539795
				MT	18.53771973	16.04142761	11.50170898
				MA	19.96718204	5.351274635	6.501708984
				MP	16.69891377	30.88781634	6.501708984
Model 53	Male	23	Left-Right	LT	-6.271793365	17.90609741	10.34912109
				LA	-6.929615843	1.994530408	2.349121094
				LP	-5.808848051	29.10392911	2.349121094
				MT	18.20578766	16.21859741	11.65509796
				MA	18.96657873	4.488701314	6.655097961
				MP	17.32849143	29.744773	6.655097961
Model 54	Male	31	Right	LT	-6.949775696	13.38156128	9.910907745
				LA	-7.58186743	1.456097497	1.910907745
				LP	-6.076699025	29.85360742	1.910907745
				MT	17.9286232	15.48144531	9.890506744
				MA	19.29936298	3.586284332	4.890506744
				MP	16.32835454	29.36843799	4.890506744
Model 55	Male	31	Left-Right	LT	-6.980163574	14.92962646	9.583488464
				LA	-7.859201044	1.77201029	1.583488464
				LP	-5.9519043	30.32082573	1.583488464
				MT	17.58082962	16.80673218	10.21900558
				MA	18.75730696	4.063027673	5.219005585
				MP	16.42078701	29.3724143	5.219005585
Model 56	Male	26	Right	LT	-5.866142273	18.28367615	10.33089447
				LA	-7.032096104	0.388513701	2.33089447
				LP	-5.025887145	31.17998611	2.33089447
				MT	17.1638298	18.32566833	10.42191696
				MA	18.22995072	5.804973429	5.421916962
				MP	15.94380845	32.65379463	5.421916962
Model 57	Male	26	Left-Right	LT	-6.425037384	16.33033752	10.15962219
				LA	-7.311868374	0.868338464	2.159622192
				LP	-5.526613007	30.46666922	2.159622192
				MT	17.32896423	18.70248413	10.89536667
				MA	18.58387369	5.82180178	5.895366669
				MP	15.96667306	32.68535735	5.895366669
Model 58	Male	26	Right	LT	-6.574863434	17.79455566	10.4603653
				LA	-7.800471526	2.018199113	2.460365295
				LP	-5.409897707	32.7903074	2.460365295
				MT	15.42767334	17.45817566	11.3729744
				MA	16.83670715	4.852240004	6.372974396
				MP	13.86301261	31.45642919	6.372974396
Model 59	Male	26	Left-Right	LT	-6.51915741	17.93173218	10.64603806
				LA	-7.748683082	1.773943306	2.646038055
				LP	-5.347928033	33.32342136	2.646038055
				MT	15.51846313	16.43035889	11.32564545
				MA	16.97706702	5.608829613	6.325645447
				MP	13.43527693	31.88572737	6.325645447

Model 60	Male	33	Left-Right	LT	-5.141426086	18.38594055	9.398273468
				LA	-5.227623116	1.230063759	1.398273468
				LP	-5.064890585	33.61887422	1.398273468
				MT	18.10092163	17.47338867	10.74182892
				MA	19.96244744	7.120039288	5.741828918
				MP	15.51703374	31.84433858	5.741828918
Model 61	Male	25	Right	LT	-5.566307068	16.36196899	9.409191132
				LA	-6.22784109	1.129028526	1.409191132
				LP	-4.941602473	30.74684956	1.409191132
				MT	17.41821671	15.25602722	11.7118187
				MA	18.81572111	4.501540632	6.711818695
				MP	15.50620298	29.96991713	6.711818695
Model 62	Male	25	Left-Right	LT	-5.596893311	17.08311462	9.681156158
				LA	-6.889317137	1.832966776	1.681156158
				LP	-4.435088569	30.79200309	1.681156158
				MT	18.05931473	15.04043579	11.54192734
				MA	19.16839634	3.461291109	6.541927338
				MP	16.68481665	29.39060799	6.541927338
Model 63	Male	25	Right	LT	-2.031757355	17.7149353	8.915805817
				LA	-1.812810188	0.765580653	0.915805817
				LP	-2.260840093	35.44891489	0.915805817
				MT	17.40576935	16.63418579	10.5669136
				MA	19.17616979	4.129886895	5.566913605
				MP	15.46408244	30.34827718	5.566913605
Model 64	Female	22	Left-Right	LT	-6.510452271	17.45310974	9.051223755
				LA	-8.005799475	2.849530188	1.051223755
				LP	-5.142592134	30.85180632	1.051223755
				MT	19.07266617	14.59960938	10.27719116
				MA	20.57728685	4.920535096	5.277191162
				MP	16.44395123	31.59150508	5.277191162
Model 65	Male	27	Right	LT	-5.047637939	17.79708862	10.7525177
				LA	-5.012669175	0.559911876	2.7525177
				LP	-5.079396951	33.45207953	2.7525177
				MT	18.26823807	18.12567139	9.438083649
				MA	19.05544863	5.54748789	4.438083649
				MP	17.37988326	32.31995479	4.438083649
Model 66	Male	27	Left-Right	LT	-5.162563324	18.52090454	10.6171608
				LA	-5.669414113	1.241772892	2.617160797
				LP	-4.712424337	33.86666506	2.617160797
				MT	17.37041092	19.23251343	9.659667969
				MA	18.20664435	4.8727655	4.659667969
				MP	16.62510425	32.03087271	4.659667969
Model 67	Male	26	Right	LT	-1.078052521	15.74301147	9.864334106
				LA	-1.331925045	1.010257007	1.864334106
				LP	-0.784893115	33.02993418	1.864334106
				MT	16.86977005	17.76011658	12.01987457
				MA	18.14510326	5.799892486	7.019874573
				MP	15.49506012	30.57002442	7.019874573
Model 68	Male	26	Left-Right	LT	-4.081863403	16.29467773	9.369537354
				LA	-4.775996629	1.203438518	1.369537354
				LP	-3.283750561	33.6465511	1.369537354
				MT	17.90004349	17.02482605	11.05999374
				MA	19.03843963	4.796267638	6.059993744
				MP	16.66124656	30.33188154	6.059993744
Model 69	Male	24	Right	LT	-5.196990967	16.84936523	10.56828308
				LA	-5.53270297	0.497523056	2.568283081
				LP	-4.897130506	31.45495243	2.568283081
				MT	18.28019333	15.59370422	11.63693237
				MA	19.83597339	4.353616603	6.636932373
				MP	16.32697598	29.70516812	6.636932373
Model 70	Male	24	Left-Right	LT	-6.239719391	16.08476257	10.31344604
				LA	-6.394853267	0.856754034	2.313446045
				LP	-6.086490391	31.12578754	2.313446045
				MT	17.50368881	17.67564392	11.94842911
				MA	19.7376859	5.022671004	6.948429108
				MP	15.3951008	29.61832157	6.948429108
