# Supplementary material for: Talar Dome Investigation and Talocrural Joint Axis Analysis Based on Three-Dimensional (3D) Models: Implications for Prosthetic Design
Source: Biomed Res Int. 2019 Nov 7;2019:8634159. doi: 10.1155/2019/8634159 (PMC6885182; doi:10.1155/2019/8634159)
Supplement: Supplementary Materials — The supplementary materials of the present study included three supplementary data files, which are referred in the manuscript and are all the raw data of this original research. [file 8634159.f1.zip › 8634159.f1/Supplementary data 3.pdf]

ID	Gender	Age	Side	R-LA	R-LP	R-MA	R-MP	Inclination Angle of Talar Dome
Reference Model	Male	33	Right	22.473737	18.717387	19.28062	20.757729	8.566
Model 1	Male	23	Right	17.318732	18.398355	17.53772	21.782868	7.634
Model 2	Male	23	Left-Right	20.378612	16.732339	17.922733	21.264819	11.496
Model 3	Female	24	Right	17.799709	19.571522	11.314658	28.156842	15.634
Model 4	Female	24	Left-Right	20.126527	26.268699	18.741775	22.28477	8.601
Model 5	Female	23	Right	14.111587	15.456499	16.742608	24.328799	17.750
Model 6	Female	23	Left-Right	17.621761	15.290661	17.275739	20.453783	12.087
Model 7	Male	25	Right	19.824512	20.305429	13.727733	22.799781	10.834
Model 8	Male	25	Left-Right	17.499345	16.845669	13.2808	27.374752	12.416
Model 9	Male	24	Right	22.070689	22.865601	18.907697	27.105788	10.942
Model 10	Male	24	Left-Right	20.465577	20.350506	19.679653	24.285791	7.442
Model 11	Female	24	Right	15.580487	24.090745	16.034591	26.327893	10.169
Model 12	Female	24	Left-Right	16.809658	17.980511	26.753863	19.941681	17.606
Model 13	Female	25	Right	17.954646	17.840518	13.86951	22.342772	13.815
Model 14	Female	25	Left-Right	15.206663	18.951557	19.533637	21.53581	16.418
Model 15	Male	25	Right	20.10273	16.307456	16.112645	24.55784	8.873
Model 16	Male	25	Left-Right	17.294555	18.569461	15.353622	22.11681	9.124
Model 17	Male	23	Right	22.822489	18.569732	14.231527	24.768824	5.119
Model 18	Male	23	Left-Right	20.146647	21.362513	13.909593	22.470794	10.469
Model 19	Male	29	Left-Right	16.515521	20.558747	16.675774	21.475659	6.868
Model 20	Male	25	Right	14.954389	19.07177	15.74062	21.288657	10.816
Model 21	Male	25	Left-Right	14.949324	13.007633	14.62671	23.896805	6.563
Model 22	Male	26	Right	21.200627	18.117627	17.948716	23.625827	5.474
Model 23	Male	26	Left-Right	21.280437	22.918483	19.230688	23.780696	6.242
Model 24	Male	24	Right	17.797769	20.058616	11.76949	17.997789	9.984
Model 25	Male	24	Left-Right	19.511442	18.319515	12.417702	19.081821	8.873
Model 26	Male	26	Left-Right	20.68175	22.764706	16.264639	24.918835	6.743
Model 27	Male	23	Right	19.313448	21.893654	22.902809	21.727752	4.332
Model 28	Male	23	Left-Right	23.023757	18.6286	24.482767	20.064685	3.915
Model 29	Male	23	Left-Right	22.449544	21.591612	16.645635	21.46382	12.463
Model 30	Male	37	Right	18.357642	15.687297	14.598669	21.515843	8.898
Model 31	Male	37	Left-Right	22.661816	19.368753	15.592783	26.487768	8.032
Model 32	Male	25	Right	22.257782	17.662411	18.579725	21.774702	8.277
Model 33	Male	25	Left-Right	17.12967	18.099563	21.64475	20.593727	8.976
Model 34	Male	24	Right	18.506517	16.548363	16.352632	20.305774	12.620
Model 35	Male	24	Left-Right	20.052707	14.859445	13.14678	20.780806	11.602
Model 36	Male	24	Right	18.658387	17.560456	13.952601	19.98776	9.168
Model 37	Male	24	Left-Right	21.579513	17.430724	14.768664	23.022761	12.948
Model 38	Female	21	Right	17.703348	19.998658	16.327802	16.420557	9.809
Model 39	Female	21	Left-Right	18.945524	17.785563	12.855502	26.145808	14.323
Model 40	Male	25	Right	19.887695	19.059667	16.998644	20.25379	7.154
Model 41	Male	25	Left-Right	18.117493	16.320694	17.089725	23.465804	12.879
Model 42	Female	25	Right	15.251705	24.407521	28.155802	24.491788	20.267
Model 43	Female	25	Left-Right	17.362601	18.354536	13.407553	31.617802	9.599
Model 44	Male	22	Right	20.522486	21.407637	17.189751	18.595792	8.768
Model 45	Male	22	Left-Right	20.601522	19.526429	15.324783	22.542793	9.954
Model 46	Male	23	Right	22.294677	16.595644	13.986819	29.071766	10.221
Model 47	Male	23	Left-Right	21.491805	19.149562	12.073617	27.058841	8.642
Model 48	Female	23	Right	17.853409	22.955488	24.672895	22.663862	13.799
Model 49	Female	23	Left-Right	17.415673	24.375587	23.931778	23.987714	13.459
Model 50	Male	27	Right	21.281566	16.031713	17.283732	24.258832	5.486
Model 51	Male	27	Left-Right	21.999761	13.900369	19.936626	19.054799	8.382
Model 52	Male	23	Right	17.672455	14.35317	14.132743	24.880728	8.770
Model 53	Male	23	Left-Right	19.851463	11.850568	16.317671	20.87369	6.078
Model 54	Male	31	Right	12.91408	21.00649	16.837815	22.04176	9.608
Model 55	Male	31	Left-Right	14.868717	18.872426	18.879632	18.424785	9.097
Model 56	Male	26	Right	24.100591	14.439337	18.290847	23.178863	8.595
Model 57	Male	26	Left-Right	18.991681	16.540608	19.249657	22.238698	9.016
Model 58	Male	26	Right	19.650479	18.139717	18.589865	22.34076	10.820
Model 59	Male	26	Left-Right	20.412458	18.892679	14.423758	26.821778	12.028
Model 60	Male	33	Left-Right	22.396543	18.503571	13.566525	23.820804	10.481
Model 61	Male	25	Right	18.530571	16.957618	14.26172	24.515885	9.891
Model 62	Male	25	Left-Right	18.6405	15.830602	16.031584	23.282714	10.315
Model 63	Male	25	Right	21.958648	23.659715	18.449778	21.685688	7.318
Model 64	Female	22	Left-Right	17.469445	15.337612	12.095519	32.063917	14.647
Model 65	Male	27	Right	22.570678	19.317786	18.383739	22.727711	3.465
Model 66	Male	27	Left-Right	22.677499	18.731546	23.19082	18.935828	5.013
Model 67	Male	26	Right	17.570502	22.683551	16.967807	19.098832	7.085
Model 68	Male	26	Left-Right	18.264653	22.858573	17.583818	20.361812	7.952
Model 69	Male	24	Right	20.718794	17.338685	15.376676	22.795747	9.057
Model 70	Male	24	Left-Right	18.495468	18.141557	19.009697	17.207817	10.597
